# Supplementary material for: Shared neural dynamics of facial expression processing
Source: Cogn Neurodyn. 2025 Mar 4;19(1):45. doi: 10.1007/s11571-025-10230-4 (PMC11880506; doi:10.1007/s11571-025-10230-4)
Supplement: Supplementary file 1 — Supplementary file1 (DOCX 1134 KB) [file 11571_2025_10230_MOESM1_ESM.docx]

# Supplementary Information

**Shared Neural Dynamics of Facial Expression Processing**

Madeline Molly ELY, Géza Gergely AMBRUS

# Classification pipelines

The figures below illustrate the classification pipelines used in this study. They detail the processes for multivariate cross-classification and representational similarity analyses applied to the electroencephalographic data.

| 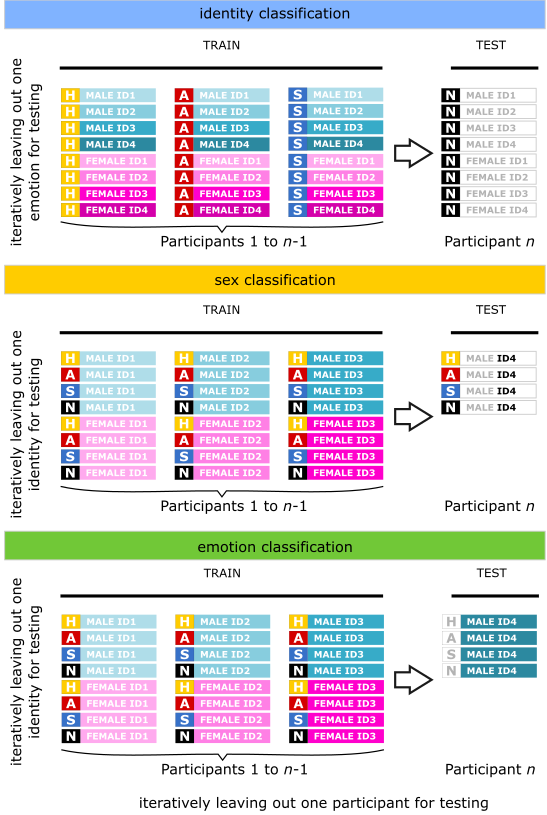 |
| --- |
| **Supplementary Information Figure S1.** In identity classification, classifiers were iteratively trained on three emotion categories and then tested on the emotion category excluded from training. For sex and emotion classifiers, training involved six distinct identities (three male and three female) and subsequent testing on one identity omitted during training. Classification accuracies were compared against chance levels (identity: 0.125, sex: 0.5, emotion: 0.25). H: happy, A: angry, S: sad, N: neutral. |

| 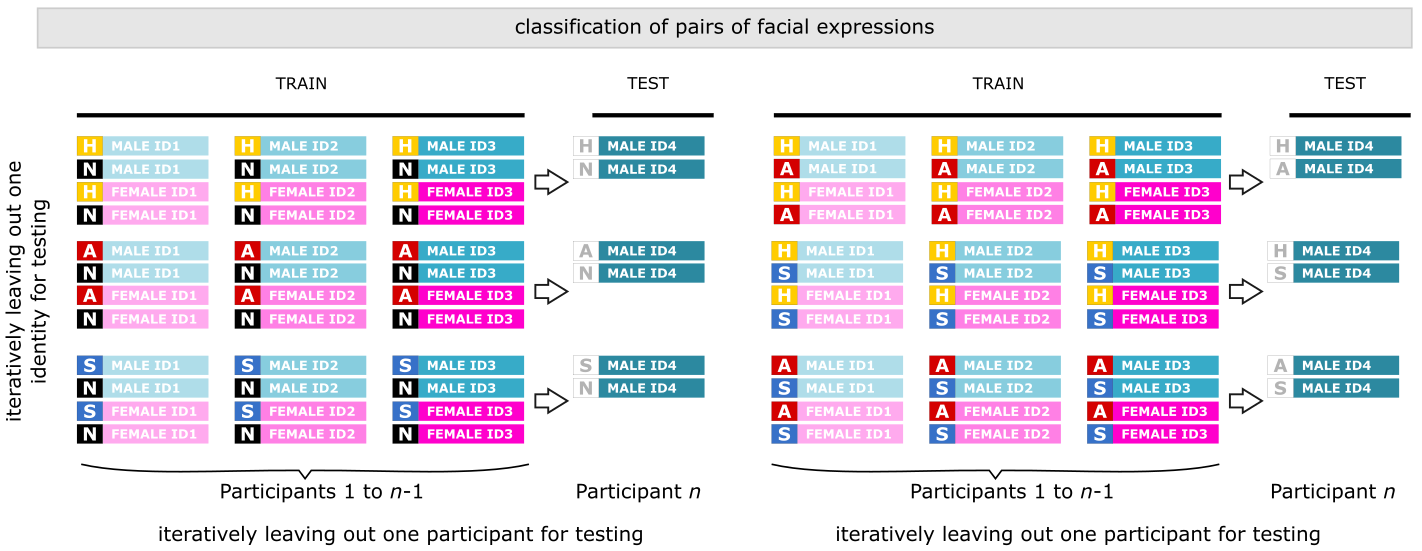 |
| --- |
| **Supplementary Information Figure S2.** The classification of pairs of emotional expressions followed a similar approach to that of emotion in general, but with two emotional expressions included at a time. Training involved six distinct identities (three male and three female) and subsequent testing on one identity omitted during training. H: happy, A: angry, S: sad, N: neutral. |

| 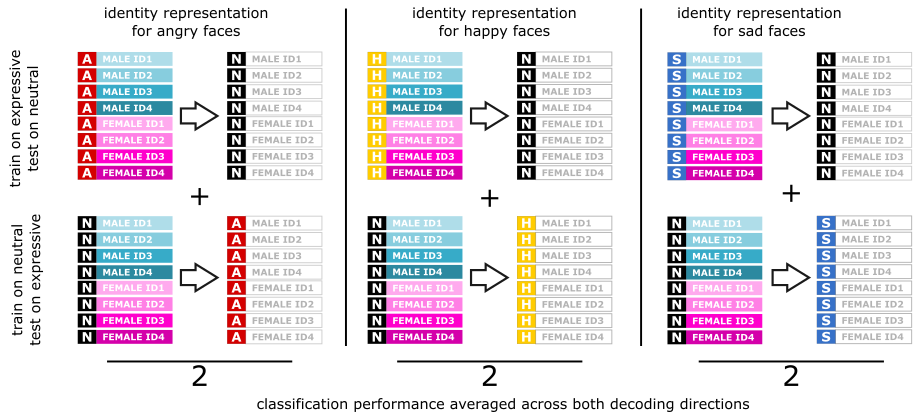 |
| --- |
| **Supplementary Information Figure S3.** To explore the temporal dynamics of face identity and sex processing across different emotional expressions, cross-classification was implemented. This method entailed training the classifier using data from trials with neutral expressions and evaluating its performance on trials featuring one of the emotional expressions, and vice versa. The obtained classification accuracies in both directions (e.g., neutral-to-angry and angry-to-neutral) were then averaged for each participant and subjected to statistical testing against chance. H: happy, A: angry, S: sad, N: neutral. |

| 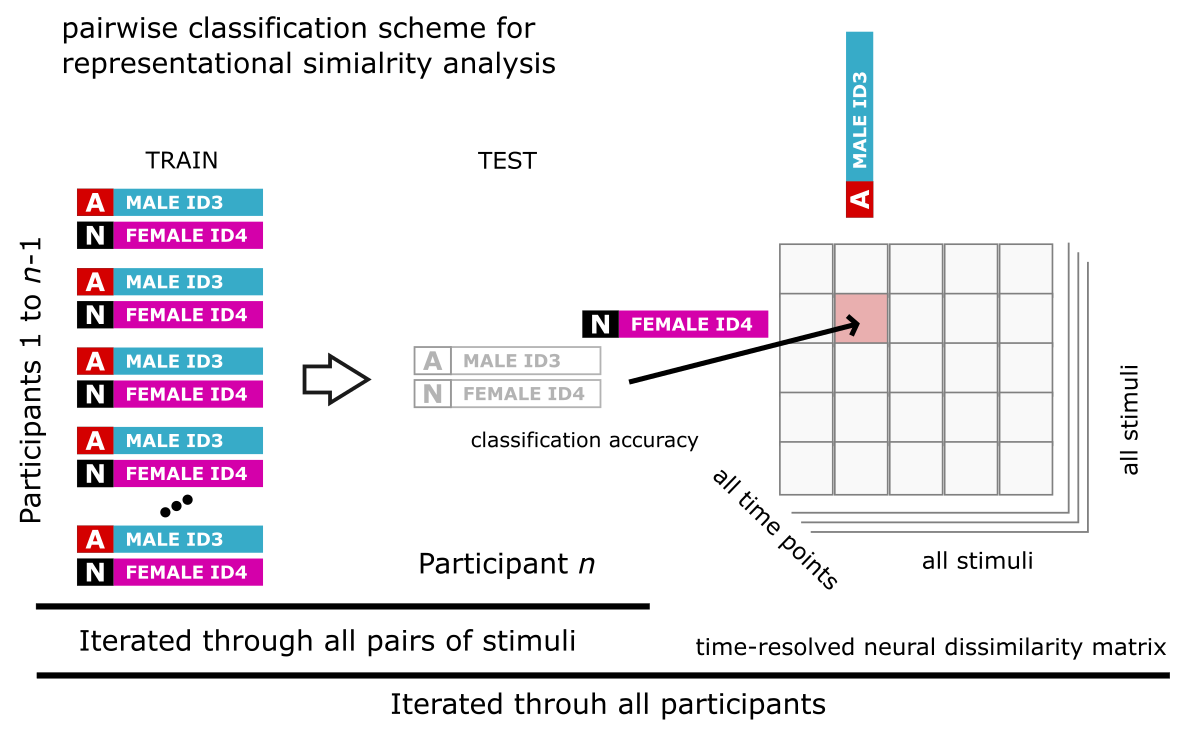 |
| --- |
| **Supplementary Information Figure S4.** **Representational similarity analyses.** Representational similarity analyses involved constructing participant-level empirical neural representational dissimilarity matrices (RDMs) in each time-point for each participant. This process was achieved through the pairwise classification of stimulus pairs, resulting in matrices with dimensions 280 by 32 by 32. The classification followed a leave-one-subject-out scheme. Subsequently, these matrices were compared to predictor representational dissimilarity matrices that modeled identity, sex, and facial expression (32 by 32 matrices), as well as pairs of expressions (16 by 16 matrices), using Spearman rank correlations. The resulting correlation values were then Fisher-transformed. H: happy, A: angry, S: sad, N: neutral. |

# Supplementary Results

| 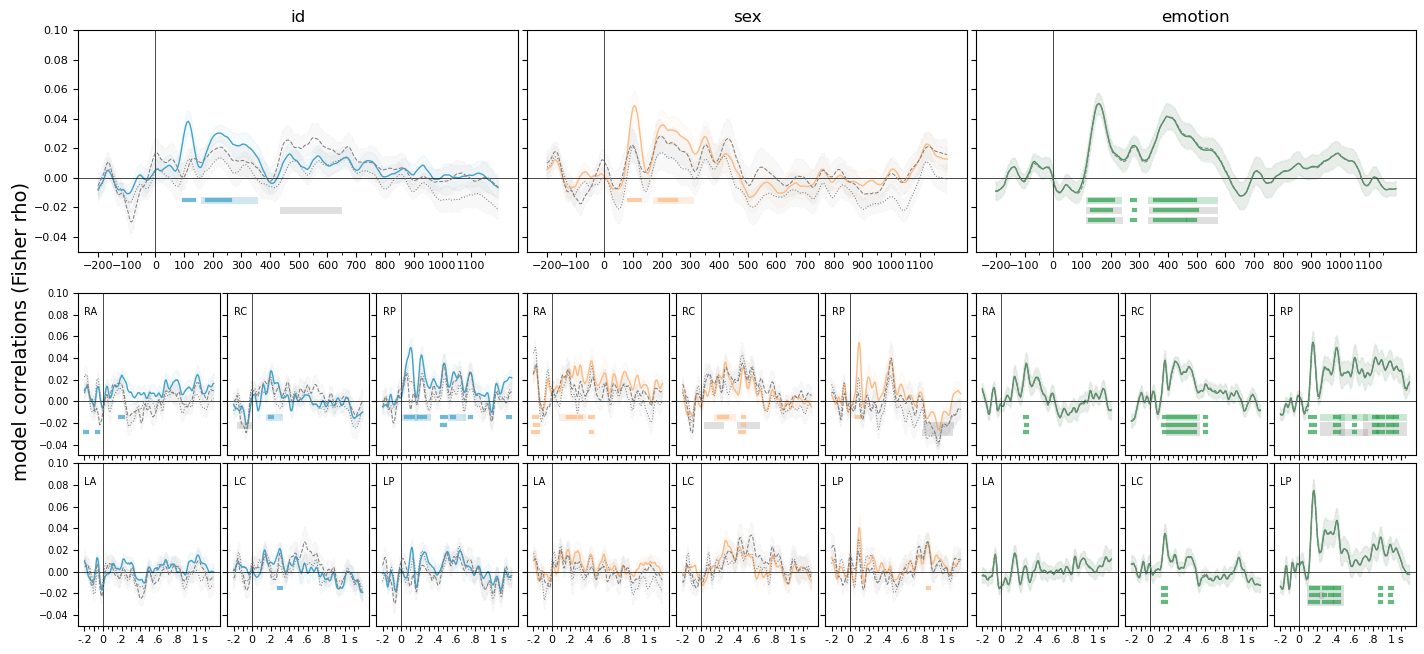 |
| --- |
| **Supplementary Figure S5. Representational Similarity Analysis for identity, sex, and facial expressions.** Results for all electrodes and pre-defined regions of interest. Neural RDMs were calculated using time-resolved, leave-one-participant-out pairwise decoding of EEG data. These matrices were then assessed against model RDMs for identity, sex, and facial expression. Solid lines represent model correlations, dashed lines illustrate results when the effects of maximum cross-correlation are controlled for, and dotted lines depict results with the effects of neural network feature distance partialled out. Light lines denote significant clusters revealed by the two-sided cluster permutation tests,*p*< 0.05; dark lines denote results of the Bayesian statistical analyses, two-sided one-sample Bayesian *t*-tests, bf >10. Error ranges denote ± SEM. Top panels: results for all electrodes. Bottom panels: results in the six pre-defined electrode clusters separately. RA/LA: right/left anterior, RC/LC: right/left central, RP/LP: right/left posterior. For detailed statistics, see **Supplementary Table 1**. |

| 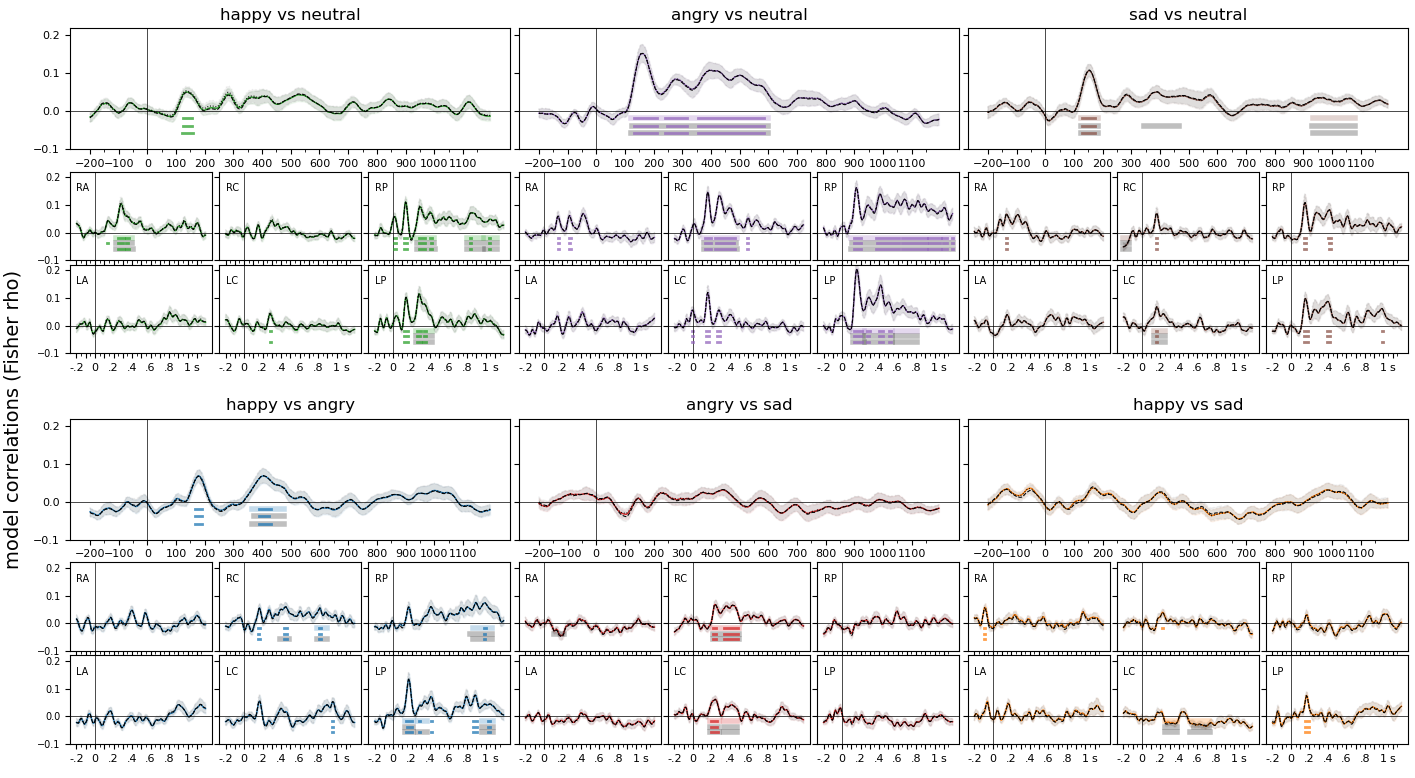 |
| --- |
| **Supplementary Figure S6. Representational Similarity Analysis for pairs of facial expressions.** Results for all electrodes and pre-defined regions of interest. Neural RDMs were calculated using time-resolved, leave-one-participant-out pairwise decoding of EEG data. These matrices were then assessed against model RDMs for pairs of facial expressions. Solid lines represent model correlations, dashed lines illustrate results when the effects of maximum cross-correlation are controlled for, and dotted lines depict results with the effects of neural network feature distance partialled out. Light lines denote significant clusters revealed by the two-sided cluster permutation tests,*p*< 0.05; dark lines denote results of the Bayesian statistical analyses, two-sided one-sample Bayesian *t*-tests, bf >10. Error ranges denote ± SEM. Top panels: results for all electrodes. Bottom panels: results in the six pre-defined electrode clusters separately. RA/LA: right/left anterior, RC/LC: right/left central, RP/LP: right/left posterior. For detailed statistics, see **Supplementary Table 1**. |
